# Effect of acute eye fatigue on cognition for young females: a pilot study

**DOI:** 10.7717/peerj.7978

**Published:** 2019-10-29

**Authors:** Ryota Akagi, Miki Tonotsuka, Ryota Horie, Kosuke Hirata, Soichi Ando

**Affiliations:** 1 College of Systems Engineering and Science, Shibaura Institute of Technology, Saitama, Japan; 2 Graduate School of Engineering and Science, Shibaura Institute of Technology, Saitama, Japan; 3 QOL Improvement and Life Science Consortium, Shibaura Institute of Technology, Saitama, Japan; 4 College of Engineering, Shibaura Institute of Technology, Tokyo, Japan; 5 Japan Society for the Promotion of Science, Tokyo, Japan; 6 Graduate School of Informatics and Engineering, The University of Electro-Communications, Tokyo, Japan

**Keywords:** Go/NoGo task, Memory recognition task, Black-and-white inverted images, Refractive error

## Abstract

The number of people suffering from eye fatigue induced by visual display terminal (VDT) viewing is expected to increase in the modern world. Eye dysfunction is suggested to induce a decrease in cognitive function, at least in the long term. Furthermore, considering other previous findings, it may be reasonable to think that acute or relatively short-term eye dysfunction attenuates cognitive function for not only older but also young individuals. Hence, clarification of the effect of eye fatigue induced by VDT viewing on cognitive performance is essential in order to maintain and/or improve our quality of life in the modern world regardless of age. The present study investigated the effect of eye fatigue induced by 1-h VDT viewing on cognitive performance, to test the hypothesis that such eye fatigue impairs cognitive performance in young individuals. A total of 19 healthy female university students voluntarily participated in this study. Before and after the 1-h VDT viewing or resting, the degree of eye fatigue and cognitive performance were evaluated. Refractive error measurement was performed to assess the degree of eye fatigue using a binocular auto refractometer, and a memory recognition task and Go/NoGo task were used to estimate cognitive performance. Response accuracy and reaction time were evaluated in the two tasks. Due to difficulty in interpreting the data of refractive error for one participant from the perspective of eye fatigue, the data for 18 participants were used for further analysis. The refractive error was significantly lower after than before the VDT viewing, but a corresponding change was not found before and after resting. Regarding cognitive performance, only the reaction time in the memory recognition task varied with the VDT viewing or resting. The reaction time was significantly longer after than before resting, without a corresponding difference before and after the VDT viewing. Thus, the 1-h VDT viewing induced eye fatigue, but relatively improved rather than attenuated reaction time in the memory recognition task. These results suggest that the effect of the increase in arousal level induced by the present VDT viewing on memory recognition compensated for the negative effect of 1-h resting of the eyes. We conclude that the acute eye fatigue induced by the 1-h VDT viewing does not have detrimental effects on cognition in young females at least under the present conditions.

## Introduction

In the modern world, the use of visual display devices such as personal computers and smartphones has become a significant part of daily life at home, at work, during leisure time and on the move. Visual display terminal (VDT) work is recognized as a high-risk factor for eye discomfort, including eyestrain (Bergqvist, 1995; Nakaishi & Yamada, 1999). Computer vision syndrome, also known as digital eyestrain, is the combination of eye and vision problems associated with VDT use (Rosenfield, 2016). Consequently, the number of people suffering from eyestrain induced by VDT work is expected to increase.

The importance of visual input for cognitive function has been suggested thus far. In an epidemiologic study (Ong et al., 2012), older individuals with visual impairment, particularly due to cataracts, were more likely to have cognitive dysfunction. They also reported that diabetic retinopathy, which is one of the major age-related eye diseases, was associated with cognitive dysfunction (Ong et al., 2012). Furthermore, another study (Spierer et al., 2016) investigating a cross-sectional correlation between vision and cognitive functions in normal aging indicated that good visual acuity and wearing glasses seem to correlate with better cognitive function. These findings suggest that eye dysfunction induces a decrease in cognitive function, at least in the long term. On the other hand, in a previous study (Park et al., 2015), young men and women watched two-dimensional and three-dimensional videos for an hour, respectively. Based on the previous results, they explained that viewing three-dimensional content requires viewers to use more cognitive resources for processing three-dimensional information compared to two-dimensional content and that eyestrain is really brain strain, indicating cumulative cognitive load. Another previous study (Daniel & Kapoula, 2019) investigating for visually normal young participants indicated an interplay between vergence accommodation conflict and cognitive load and the link between cognition and high quality of single binocular vision. In addition, as shown in a recent review (Orru & Longo, 2019), visual input relates to working memory as well as auditory input, which is one of premise of Cognitive Load Theory. Considering these findings, it may be reasonable to think that acute or relatively short-term eye dysfunction attenuates cognitive function for not only older but also young individuals. Therefore, clarification of the effect of eyestrain induced by VDT work on cognitive performance is essential in order to maintain and/or improve our quality of life in the modern world regardless of age.

Digital eyestrain or computer vision syndrome involves a group of ocular and nonocular symptoms among the users of visual display units; of those, ocular symptoms are more common (Blehm et al., 2005). In accordance with a latest review article (Coles-Brennan, Sulley & Young, 2019), although the term “computer vision syndrome” has been widely used in the literature, “digital eyestrain” seems a more appropriate term because many other digital devices are now in common use. Meanwhile, there is likely to be a lot of overlap between eyestrain, eye fatigue, visual fatigue and asthenopia. A previous study (Wang et al., 2018) regarded eye fatigue as the VDT-caused fatigue and was only interested in the internal change of eye. Based on this, they provided a novel definition of eye fatigue. The current study followed this definition.

A traditional method to assess eye fatigue is using questionnaire (Nahar et al., 2011; Wang et al., 2018) because patients with or workers complaining of eye fatigue are classified according to their subjective complaints. That is, this method cannot evaluate eye fatigue objectively. A previous study (Suh et al., 2012) paid attention to nearwork-induced transient myopia and investigated the degree of eye fatigue using a change in refractive error (spherical equivalent value) before and after a task. As a result, the task-induced myopic shift was found, implying that the refractive error is useful for assessing the degree of eye fatigue. In another study (Collier & Rosenfield, 2011) investigating the degree of eye fatigue during a VDT work (a 30-min reading task using a personal computer), the refractive error gradually shifted to myopia without statistical significance; however, this study did not compare the results of a control condition or group. Correspondingly, there is still a possibility that the longer VDT work compared to the previous study can significantly shift the refractive error to myopia.

In the present study, the effect of eye fatigue induced by 1-h VDT viewing on cognitive performance was investigated in young individuals. We hypothesized that, (1) the 1-h VDT viewing results in eye fatigue and (2) the 1-h VDT viewing-induced eye fatigue impairs cognitive performance. The purpose of the present study was to test the hypothesis.

## Materials and Methods

### Participants

After providing written informed consent, 19 healthy university female students (age: 21 ± 1 year, height: 159.9 ± 4.7 cm, body mass: 54.0 ± 9.0 kg; mean ± standard deviation (SD)) voluntarily participated in this study. They were free of cardiovascular and eye diseases, and used a personal computer and a smartphone regularly. Seven participants lived without contact lenses or glasses, and performed the tasks with their naked eyes. Given that refractive error measurement is usually conducted with a patient’s naked eyes and glasses are easier to put on and take off than contact lenses, the other participants who habitually used contact lenses or glasses performed the tasks except for the corresponding measurement with their glasses. In doing so, the effect of differences in visual parameters characterizing the accommodative and binocular function between wearing contact lenses and glasses (Jiménez et al., 2011) on the current study was removed. All of the corresponding participants were used to wearing glasses. This study was approved by the Ethics Committee of the Shibaura Institute of Technology (18-011) and conducted according to the Declaration of Helsinki.

### Experimental procedures

**Figure 1:**
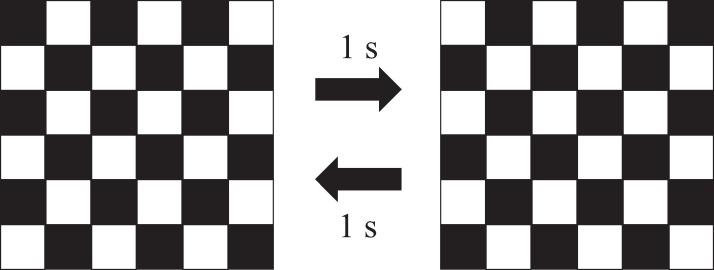
Square lattice and black-and-white inverted images changing every second.

Two conditions were used in the present study. In one condition, participants lay prone on a bed featuring an elliptical hole (~200 × 120 mm) and continuously watched square lattice and black-and-white inverted images that changed every second (Fig. 1) displayed on a personal computer screen (TPN-C126, HP Japan, Japan; size: 15 inch, resolution: 1,366 × 768, brightness: max) for 1 h (VDT viewing condition). The corresponding images were used to induce eye fatigue in accordance with a previous study (Chen et al., 2015) and were made using Microsoft Office PowerPoint 2016 (Microsoft, Redmond, WA, USA). They were saved as a MP4 file, and were played using Windows Media Player (ver. 12; Microsoft, Redmond, WA, USA). The screen was set under the bed and the participants watched from the elliptical hole so that the viewing distance (~300 mm) was kept constant during the VDT viewing condition without fatigue or discomfort in the participants’ body parts other than the eyes (e.g., shoulder, neck and lower back). In the other condition, they lay supine on a bed and rested their eyes using a blindfold for 1 h (resting condition). An investigator spoke to the participants regularly to prevent them from sleeping in the both conditions. Based on refractive error measurements and conducting cognitive tasks (see below) before and after these actions (pre-test and post-test, respectively), the degree of eye fatigue and cognitive performance were assessed. The two conditions were randomly performed for each participant on two consecutive days. The experiments were carried out by turning on the light in the experimental room.

On the first day, sighting eye dominance was assessed by variations of both the Porta and Miles tests. First, participants were asked to move a paper having a three-cm diameter hole to their face, and the eye which they moved this hole closer to was taken as the dominant eye. Second, participants were asked to extend one arm to point at a landmark object in front of them a few meters away with both eyes open, and to close each eye alternately. The differences in the distance between the landmark object and each eye were evaluated, and the open eye with the smaller difference was taken as the dominant eye. If the results of these methods were different, a third method was performed: participants were asked to match a vertical line described on a whiteboard with a slim pen held in the hand with both eyes open, and to close each eye alternately. The differences in distance between the line and the pen were evaluated, and the open eye with the smaller difference was taken as the dominant eye. Subsequently, the participants were familiarized with two cognitive tasks before starting the experiment: the memory recognition task (Lefferts et al., 2016) and the Go/NoGo task (Ando et al., 2015; Komiyama et al., 2017; Wessel, 2018). The former requires memory recognition (Lefferts et al., 2016), and the latter requires executive function, including selective attention, response inhibition and interference control (Pontifex et al., 2009; Chaddock et al., 2011). The specific procedures are described below.

On each day, the participants lay in a prone position on the bed, wore a blindfold, and closed their eyes for 10 min prior to exposure to alleviate previous induced visual fatigue or cognitive load due to daily life’s screen uses (smartphones and/or personal computers) in accordance with a previous study (Shieh & Chen, 1997). The degree of eye fatigue and cognitive performance were then evaluated randomly (pre-test). As described in the Introduction, the refractive error measurement was performed to evaluate the degree of eye fatigue using a binocular auto refractometer (WV-500; Grand Seiko, Tokyo, Japan). The participants were asked to sit on a chair without corrective lenses, to set their chin on a chin rest of the refractometer and to touch their forehead on the bar of the refractometer. The refractive error for the dominant eye was measured once in 0.125 D steps while the participants continued to look ahead. When performing the cognitive tasks, the participants sat on a chair and faced a personal computer screen placed on a desk. First, they memorized 30 Japanese words displayed on the personal computer screen at a rate of every 2 s (i.e., total time: 60 s) before performing the memory recognition task. Second, they conducted the Go/NoGo task. When the participants were ready, they clicked the left button of a PC mouse with their right hand to start the task. A black screen was displayed for 2.5 s and then green (RGB value: 0, 255, 0) square for 1 s. Next, a red (255, 0, 0), blue (0, 0, 255), purple (255, 0, 255) or orange (255, 223, 0) square was superimposed for 1 s. The size of the colored square was 100 × 100 mm. When the participants judged that a red or blue square was displayed, they clicked the left button of the mouse as quickly as possible. When a purple or orange square was displayed, they waited for the next question without doing anything. Thereafter, a black screen was displayed again. This routine was repeated 60 times until each color square had been displayed 15 times, in random order. Finally, the memory recognition task was performed. The participants clicked the left mouse button to start the task and a black screen was displayed for 1 s. Next, a word was displayed for 2 s. Here, if they judged that they had memorized the word before the task, they clicked the left mouse button as quickly as possible, or otherwise waited for the next question without doing anything. Afterward, a black screen was displayed again. This routine was repeated 60 times. In other words, 60 different words including 30 distracters, appeared once. In each trial of both tasks, the PC recorded whether the answer was correct or incorrect and the reaction time of each trial from when the word or colored square was displayed to when the participants clicked the left mouse button. When the participants did not click the mouse, the reaction time was not recorded regardless of whether the answer was correct or incorrect. The total time for the two cognitive tasks was about 10 min. These cognitive tasks were programed and conducted using a stimulus delivery and experiment control program for neuroscience (Presentation ver. 19; NeuroBehavioral Systems, Berkeley, CA, USA) which was installed in the aforementioned personal computer (TPN-C126; HP Japan, Tokyo, Japan; size: 15 inch, resolution: 1,366 × 768, brightness: max). On the second day, the participants spent 1 h under the other condition (the VDT viewing condition or the resting condition), and the same measurements to evaluate the degree of eye fatigue and cognitive performance were repeated (post-test).

### Data analyses

Emmetropia has often been classified as a spherical equivalent = 0 D (Yeh et al., 2019) or between −0.50 and 0.50 D (Kubai et al., 2013; Guo et al., 2017), indicating that the farther the spherical equivalent is from 0, the farther eye refraction is from normal. In the present study, the VDT viewing was expected to result in the myopic shift (i.e., negative direction). The data for 18 participants showed negative values of refractive error for all measurements, but the data for one participant included both positive and negative values of refractive error. It was difficult to interpret the result of a change in refractive error straddling the value “0” between pre- and post-tests from the perspective of eye fatigue, so the data of refractive error measurement and cognitive performance tasks for that participant were excluded from further analysis.

Response accuracy and reaction time in each task were used to evaluate cognitive performance. We calculated the response accuracy as follows: Response accuracy (%) = Number of correct trials/60 × 100. The recorded reaction time was averaged in the correct trials for use in further analyses. In other words, the recorded reaction times in incorrect trials were not considered.

### Statistical analyses

The Kolmogorov–Smirnov test was used to check normality of the data. Some data of the memory recognition task and Go/NoGo task were not normally distributed. Considering the present experimental design, a two-way analysis of variance (ANOVA) (i.e., a parametric test) was required. Therefore, all data obtained in the two tasks were log-transformed before analyses. For ease of interpretation, the related data of these tests are presented as means ± SDs of raw data, as with the data of the refractive error measurement.

A two-way ANOVA with two within-group factors (time (pre-test and post-test), condition (resting condition and VDT viewing condition)) was used to evaluate changes induced by the VDT viewing in the refractive error measurement and in the response accuracy and reaction time for the tasks of cognitive performance. When a significant interaction was detected, additional ANOVA with Bonferroni correction was performed. On the other hand, for each variable, we calculated the amount of change from pre-test to post-test and evaluated the difference in the corresponding amount of change between the conditions using a paired *t*-test. When the difference was significant, Cohen’s *d* was also calculated as an effect size. In the current study, the *d* values were interpreted as 0.20–0.49, 0.50–0.79 and ≥0.80 for small, moderate and large effects, respectively (Cohen, 1988, 1992).

Statistical analyses were performed using SPSS (version 25.0; IBM, Armonk, NY, USA). Significance was set at *P* < 0.05. When the results of the two-way ANOVA are presented, partial η^2^ (η_p_^2^) is shown as an index of the effect size with the *P*-value.

## Results

**Figure 2:**
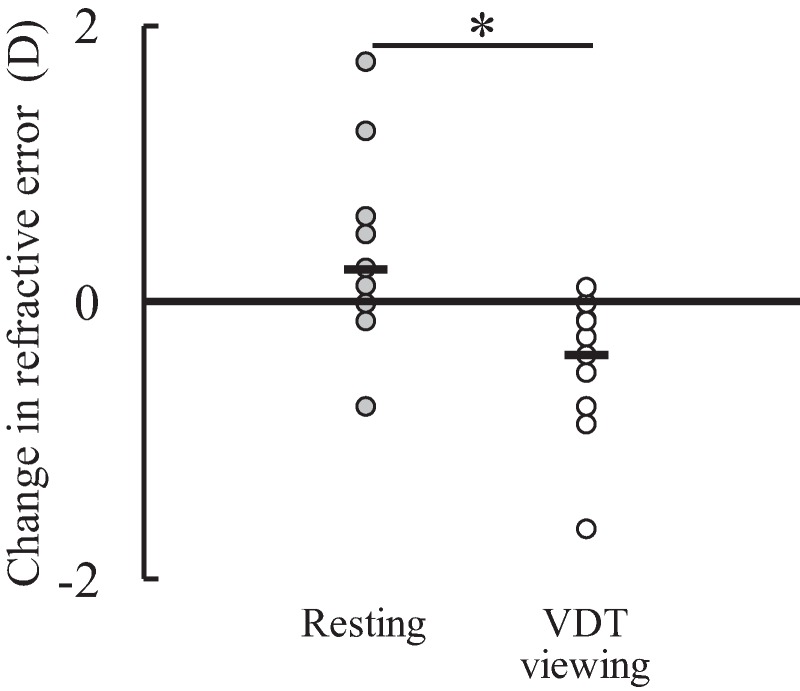
Individual data of the amount of change in refractive error from pre-test to post-test in the resting and visual display terminal (VDT) viewing conditions (*n* = 18). Black bar shows the mean value. *Indicates a significant difference between the conditions.

In the refractive error measurements, there was a significant time × condition interaction (*F*(1,17) = 17.291, *P* = 0.001, η_p_^2^ = 0.504). There were no significant differences between the conditions either at pre-test (resting: −3.33 ± 2.46 D, VDT viewing: −3.36 ± 2.36 D; *F*(1,17) = 0.006, *P* = 0.941, η_p_^2^ < 0.001) or post-test (resting: −3.10 ± 2.26 D, VDT viewing: −3.75 ± 2.35 D; *F*(1,17) = 3.977, *P* = 0.062, η_p_^2^ = 0.190). The refractive error at post-test was significantly lower than that at pre-test in the VDT viewing condition (*F*(1,17) = 12.520, *P* = 0.003, η_p_^2^ = 0.424), but the corresponding difference was not significant in the resting condition (*F*(1,17) = 3.305, *P* = 0.087, η_p_^2^ = 0.163). There was a significant difference in the amount of change in the refractive error from pre-test to post-test between the resting (0.24 ± 0.55 D) and VDT viewing (−0.39 ± 0.47 D) conditions (*P* = 0.001, *d* = 1.23: large effect) (Fig. 2).

**Figure 3:**
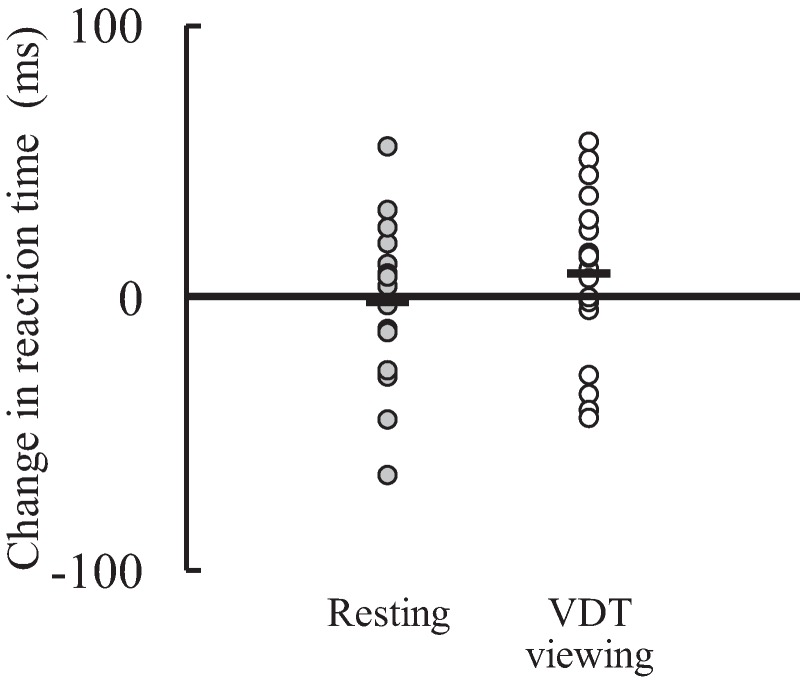
Individual data of the amount of change in reaction time in the Go/NoGo task from pre-test to post-test in the resting and visual display terminal (VDT) viewing conditions (*n* = 18). Black bar shows the mean value.

**Figure 4:**
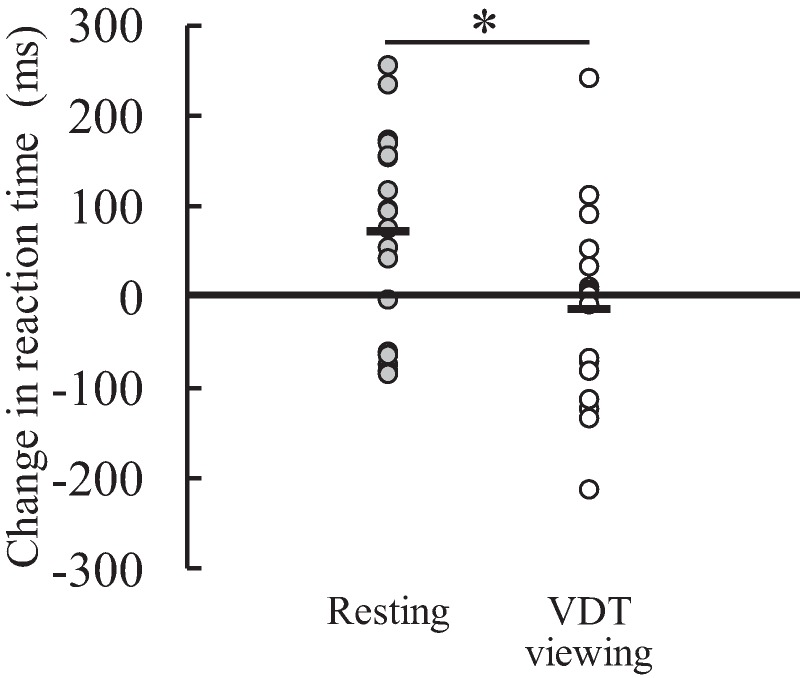
Individual data of the amount of change in reaction time in the memory recognition task from pre-test to post-test in the resting and visual display terminal (VDT) viewing conditions (*n* = 18). Black bar shows the mean value. *Indicates a significant difference between the conditions.

The response accuracy of the Go/NoGo task were 99.3% ± 0.9% in the VDT viewing condition and 99.3% ± 1.2% in the resting condition at pre-test, and 99.6% ± 0.9% in the VDT viewing condition and 99.0% ± 1.3% in the resting condition at post-test. The time × condition interaction (*F*(1,17) = 1.677, *P* = 0.213, η_p_^2^ = 0.090), main effect of time (*F*(1,17) < 0.001, *P* = 0.994, η_p_^2^ < 0.001), and main effect of condition (*F*(1,17) = 3.295, *P* = 0.087, η_p_^2^ = 0.162) were not significant. There was no significant difference in the amount of change in the response accuracy of the Go/NoGo task from pre-test to post-test between the resting (−0.3% ± 1.4%) and VDT viewing (0.3% ± 1.0%) conditions (*P* = 0.202). Similarly, the corresponding interaction (*F*(1,17) = 0.462, *P* = 0.506, η_p_^2^ = 0.026) and main effects (time: *F*(1,17) = 0.776, *P* = 0.391, η_p_^2^ = 0.044; condition: *F*(1,17) = 3.712, *P* = 0.071, η_p_^2^ = 0.179) for the reaction time of the Go/NoGo task (pre-test: 365 ± 59 ms (resting) and 370 ± 37 ms (VDT viewing), post-test: 363 ± 46 ms (resting) and 379 ± 50 ms (VDT viewing)) and the corresponding amount of change in the reaction time of the Go/NoGo task (resting: −2 ± 29 ms, VDT viewing: 9 ± 31 ms; *P* = 0.506) (Fig. 3) were also not significant. Regarding the response accuracy in the memory recognition task (pre-test: 76.9% ± 8.9% (resting) and 76.1% ± 6.3% (VDT viewing), post-test: 70.6% ± 9.7% (resting) and 73.3% ± 7.3% (VDT)), there was no significant time × condition interaction (*F*(1,17) = 1.143, *P* = 0.300, η_p_^2^ = 0.063) or main effect of condition (*F*(1,17) = 0.347, *P* = 0.564, η_p_^2^ = 0.020) with no significant difference in the amount of change from pre-test to post-test between the resting (−6.3% ± 8.3%) and VDT viewing (−2.8% ± 9.8%) conditions (*P* = 0.300). A main effect of time for the response accuracy was significant, with a greater value at pre-test than at post-test (*F*(1,17) = 11.731, *P* = 0.003, η_p_^2^ = 0.408). For the reaction time in the memory recognition task (pre-test: 784 ± 129 ms (resting) and 817 ± 161 ms (VDT viewing), post-test: 855 ± 113 ms (resting) and 804 ± 133 ms (VDT viewing)), a significant time × condition interaction was found (*F*(1,17) = 7.037, *P* = 0.017, η_p_^2^ = 0.293). The difference between the conditions was significant at post-test (resting condition > VDT viewing condition; *F*(1,17) = 7.199, *P* = 0.016, η_p_^2^ = 0.297) but not at pre-test (*F*(1,17) = 1.444, *P* = 0.246, η_p_^2^ = 0.078). The reaction time was significantly longer at post-test than at pre-test in the resting condition (*F*(1,17) = 7.702, *P* = 0.013, η_p_^2^ = 0.312), but the corresponding difference between pre-test and post-test was not significant in the VDT viewing condition (*F*(1,17) = 0.168, *P* = 0.687, η_p_^2^ = 0.010). A significant difference in the corresponding amount of change from pre-test to post-test between the resting (71 ± 111 ms) and VDT viewing (−13 ± 105 ms) conditions was found (*P* = 0.017, *d* = 0.077: moderate effect) (Fig. 4).

## Discussion

In the present study, the refractive error was significantly lower at post-test than at pre-test in the VDT viewing condition, in contrast to the resting condition, with the greater change from pre-test to post-test in the VDT viewing condition compared with the resting condition (Fig. 2). These results indicate that the 1-h VDT viewing induced eye fatigue. The reaction time in the memory recognition task was significantly longer at post-test than at pre-test in the resting condition, but there was no corresponding difference in the VDT viewing condition. Furthermore, the corresponding amount of change from pre-test to post-test was significantly greater in the resting condition than in VDT viewing condition (Fig. 4). Thus, cognitive performance seems to have deteriorated in the resting condition but not in the VDT viewing condition. In the present study, it was hypothesized that (1) the 1-h VDT viewing results in eye fatigue and (2) the 1-h VDT viewing-induced eye fatigue impairs cognitive performance. The present results support the former hypothesis but refute the latter one.

In the VDT viewing condition, the amount of change in the refractive error from pre-test to post-test was −0.39 ± 0.47 D (Fig. 2). In a previous study (Suh et al., 2012), the change in refractive error from before to after watching 3-h two-dimensional and three-dimensional movies were reported to be −0.10 and −0.36 D, respectively. Comparing the current results with the previous ones, although it is difficult to objectively evaluate the degree of eye fatigue in each case and there may be a better indicator for eye fatigue than refractive error, it is reasonable to assume that eye fatigue was induced by the 1-h VDT viewing to a certain degree in the present study as expected. The *d* value for the corresponding amount of change (1.23: large effect) also support this supposition.

There was a significant time × condition interaction for the reaction time in the memory recognition task. The reaction time was not significantly changed from pre-test to post-test in the VDT viewing condition. In contrast, the reaction time was significantly longer at post-test than at pre-test in the resting condition. Consequently, a significantly longer reaction time in the resting condition was observed than in the VDT viewing condition at post-test, and the corresponding amount of change from pre-test to post-test was significantly greater in the resting condition than in the VDT viewing condition (Fig. 4). That is, the 1-h VDT viewing improved rather than impaired cognitive performance at least under the present conditions, despite the induction of eye fatigue. This result was opposite to our expectation, but can be explained by the inverted-U relationship between arousal level and performance (Schmidt & Lee, 2000). The longer reaction time in the memory recognition task at post-test than at pre-test in the resting condition indicates that the 1-h eye rest period negatively affected memory recognition. In contrast, stimulation of the eyes and brain by the 1-h VDT viewing might have induced an increase in arousal level. If the degree of the corresponding increase was appropriate to enhance cognitive performance, it is not surprising that the cognitive performance improved after the 1-h VDT viewing compared to the resting condition. In this case, the effect of the increase in arousal level induced by the VDT viewing on memory recognition compensated for the negative effect of the 1-h rest. Here, we also need to pay attention to an effect of boredom on the corresponding results. In the present study, the participants seemed to feel boredom in both conditions, but it is not clear whether the degree of boredom was different between them. A previous study (Danckert et al., 2018) suggests that restlessness and sleepiness may reflect oscillations between high and low arousal states that have in common the desire to escape the situation. Similarly, another previous study (Malkovsky et al., 2012) found that boredom proneness was correlated with attention-related cognitive errors. Therefore, we cannot rule out the possibility that boredom affected the degree of arousal level as described above. In the future, it is important to make the experimental design that can reduce the effect of boredom on the interpretation of the experimental results as much as possible in order to argue the effect of eye fatigue induced by the VDT viewing on cognitive performance in more detail compared to the present study. Another possible reason for the results of the reaction time in the memory recognition task is an effect of a difference in brightness. According to a previous study (Chawla et al., 2019), if there is a large difference between screen brightness and ambient light, the demand on focusing mechanism is increased when the reader changes focus from the monitor to other areas of the room. Although we do not know how long the effect of the corresponding difference will continue, there was certainly a big difference in the brightness between when the participants rested their eyes using a blindfold for 1 h and when they performed the measurements using a personal computer at post-test. This may affect the result of the lower cognitive performance in the resting condition than in the VDT viewing condition.

In contrast to the results of the memory recognition task, those of the Go/NoGo task did not differ between the two conditions. The Go/NoGo task requires executive function, including selective attention, response inhibition and interference control (Pontifex et al., 2009; Chaddock et al., 2011), as described in “Material and Methods,” and several fields in the prefrontal cortex are considered to be activated in relation to this test (Kawashima et al., 1996; Watanabe et al., 2002), while tasks requiring memory recognition (Lefferts et al., 2016) seem to be affected by activation of the hippocampus (Rolls et al., 1993; Papanicolaou et al., 2002). Hence, the discrepancy between the results of the cognitive tasks may imply that the effect of the 1-h VDT viewing varies among regions of the brain. Another factor may be the difference in difficulty level between the tasks. The response accuracy of the Go/NoGo task was approximately 100%, regardless of time or condition, in contrast to that of the memory recognition task (70.6–76.9%), indicating that the former task was much easier than the latter task for the participants in this study. The present results may suggest that the effect of eye fatigue on cognitive performance is dependent on task complexity.

We would like to discuss some limitations of this study. The first limitation is that the current results were based only on females. Basically, it is considered that there is no sex difference in cognitive performance such as used here (Eliot, 2019). Therefore, the current results could apply to young males (i.e., young individuals). The second limitation is that an effect of presence or absence of myopia correction on the current results was not considered. With regard to vision correction, the present experiment was designed so that participants could work on the cognitive tasks under the same conditions as in daily life. Consequently, some of the participants may have participated in the experiment with their naked eyes, even though their myopia should be corrected from a medical point of view. In this case, there is a possibility that eye fatigue was not induced properly after the 1-h VDT viewing for such participants. In the current study, however, the presence or absence of myopia correction was the same between the VDT viewing and resting conditions for each participant. Considering the experimental design of the present study, therefore, the existence of uncorrected myopia seems to have a small effect on the interpretation of the current results. The third limitation is that the 1-h VDT viewing induced eye fatigue to a certain degree, but it is not certain whether the degree of eye fatigue induced was greater than that occurring in daily life. As described in the “Introduction,” the use of visual display devices has become a significant part of daily life at home, at work, during leisure time and on the move. Correspondingly, we spend a long time doing VDT viewing in our daily lives, indicating overuse of our eyes. In other words, the degree of eye fatigue induced by the 1-h VDT viewing in the present study may have been smaller than that during daily life. If so, there is a possibility that the present results depend on the content of VDT viewing. However, the *d* value for the amount of change in the refractive error from pre-test to post-test (1.23) was interpreted as large effect, suggesting that eye fatigue was fully induced by the 1-h VDT viewing in the current study. The fourth limitation is that participants lay prone on a bed during the 1-h VDT viewing condition in order not to feel fatigue or discomfort in their body parts other than the eyes (e.g., shoulder, neck and lower back). However, it is difficult to say that this posture is common during actual VDT viewing. The last limitation is that we assessed only memory function and executive function in the present study. Hence, investigating the effects of VDT viewing-induced eye fatigue on other aspects of cognitive function should also be done in the future.

## Conclusions

The present study investigated whether eye fatigue induced by 1-h VDT viewing negatively affects cognitive performance in young females. Although the 1-h VDT viewing induced eye fatigue to a certain degree, as expected, it did not attenuate but relatively improved reaction times in the memory recognition task. These results suggest that the acute eye fatigue induced by 1-h VDT viewing does not have detrimental effects on cognition in young females at least under the present conditions.

## Supplemental Information

10.7717/peerj.7978/supp-1 Supplemental Information 1 Raw data.

## Additional Information and Declarations

### Data Availability

The following information was supplied regarding data availability: The raw data is available in Figs. 2–4 and as a Supplemental File.
